# ChatGPT’s Attitude, Knowledge, and Clinical Application in Geriatrics Practice and Education: Exploratory Observational Study

**DOI:** 10.2196/63494

**Published:** 2025-01-03

**Authors:** Huai Yong Cheng

**Affiliations:** 1 Minneapolis VA Health Care System Minneapolis, MN United States

**Keywords:** ChatGPT, geriatrics attitude, ageism, geriatrics competence, geriatric syndromes, polypharmacy, falls, aging, older adults

## Abstract

**Background:**

The increasing use of ChatGPT in clinical practice and medical education necessitates the evaluation of its reliability, particularly in geriatrics.

**Objective:**

This study aimed to evaluate ChatGPT’s trustworthiness in geriatrics through 3 distinct approaches: evaluating ChatGPT’s geriatrics attitude, knowledge, and clinical application with 2 vignettes of geriatric syndromes (polypharmacy and falls).

**Methods:**

We used the validated University of California, Los Angeles, geriatrics attitude and knowledge instruments to evaluate ChatGPT’s geriatrics attitude and knowledge and compare its performance with that of medical students, residents, and geriatrics fellows from reported results in the literature. We also evaluated ChatGPT’s application to 2 vignettes of geriatric syndromes (polypharmacy and falls).

**Results:**

The mean total score on geriatrics attitude of ChatGPT was significantly lower than that of trainees (medical students, internal medicine residents, and geriatric medicine fellows; 2.7 vs 3.7 on a scale from 1-5; 1=*strongly disagree*; 5=*strongly agree*). The mean subscore on positive geriatrics attitude of ChatGPT was higher than that of the trainees (medical students, internal medicine residents, and neurologists; 4.1 vs 3.7 on a scale from 1 to 5 where a higher score means a more positive attitude toward older adults). The mean subscore on negative geriatrics attitude of ChatGPT was lower than that of the trainees and neurologists (1.8 vs 2.8 on a scale from 1 to 5 where a lower subscore means a less negative attitude toward aging). On the University of California, Los Angeles geriatrics knowledge test, ChatGPT outperformed all medical students, internal medicine residents, and geriatric medicine fellows from validated studies (14.7 vs 11.3 with a score range of –18 to +18 where +18 means that all questions were answered correctly). Regarding the polypharmacy vignette, ChatGPT not only demonstrated solid knowledge of potentially inappropriate medications but also accurately identified 7 common potentially inappropriate medications and 5 drug-drug and 3 drug-disease interactions. However, ChatGPT missed 5 drug-disease and 1 drug-drug interaction and produced 2 hallucinations. Regarding the fall vignette, ChatGPT answered 3 of 5 pretests correctly and 2 of 5 pretests partially correctly, identified 6 categories of fall risks, followed fall guidelines correctly, listed 6 key physical examinations, and recommended 6 categories of fall prevention methods.

**Conclusions:**

This study suggests that ChatGPT can be a valuable supplemental tool in geriatrics, offering reliable information with less age bias, robust geriatrics knowledge, and comprehensive recommendations for managing 2 common geriatric syndromes (polypharmacy and falls) that are consistent with evidence from guidelines, systematic reviews, and other types of studies. ChatGPT’s potential as an educational and clinical resource could significantly benefit trainees, health care providers, and laypeople. Further research using GPT-4o, larger geriatrics question sets, and more geriatric syndromes is needed to expand and confirm these findings before adopting ChatGPT widely for geriatrics education and practice.

## Introduction

### Background

ChatGPT stands for Chat Generative Pretrained Transformer and was developed by an artificial intelligence (AI) research company, OpenAI. It is an AI chatbot technology that can process our natural human language and generate a response. ChatGPT was released to the public by OpenAI on November 30, 2022, and surpassed 1 million users in just 5 days. This performance set a record for the second fastest-growing user base after that of Threads. ChatGPT currently has >180 million users (as of October 26, 2024) [1]. ChatGPT users have increased exponentially, and it has been increasingly used in medicine, resulting in 4625 publications on PubMed (as of October 26, 2024) [2]. Notably, ChatGPT was not programmed originally to be used for medical education and clinical application [1] despite reports of its wide application for medical education [3-7] and clinical practice [5-12]. ChatGPT has been studied in multiple specialties and subspecialties, such as psychiatry [13], radiology [14], and cardiology [15]. Its rapid progress in such a brief time has raised challenges, concerns, and limitations, including privacy, ethnic and racial bias, and legal risks [16-21].

Any application of ChatGPT to medical education and clinical practice could be promising but needs to be well designed and investigated. In the context of a growing aging population, geriatrics education, geriatrics workforces, and geriatrics practice have been facing significant challenges for the last 4 decades [22-26]. ChatGPT could offer an opportunity to improve geriatrics education and clinical practice [27-30]. Preliminary studies have shown promise [27-30]. For example, older adults often have polypharmacy issues [31], and providers review and deprescribe medications [32]. A specifically trained large language model may provide useful clinical support in polypharmacy management for primary care physicians [29]. ChatGPT has scored better on general and theoretical questions in geriatrics than on complex decisions and end-of-life situations, with the lowest scores related to diagnosis and performing complex tests [30]. Overall, the application of ChatGPT to geriatrics education and clinical practice is sporadic and needs to be expanded and further investigated. Therefore, this study was designed to determine whether we can trust ChatGPT to be used in geriatrics education and clinical practice using 3 distinct approaches.

The first approach was to evaluate any age-biased outputs generated by ChatGPT. The aging narrative spans >210 years [33]. The term ageism, coined by Robert Butler in 1969, refers to age-based stereotyping, prejudice, and discrimination [34]. Self-perception of aging is another emergent concept related to ageism [35]. Ageism is global and prevalent in daily life [34,36,37], health care systems [34,38], and the news media [39,40]. It is present in various medicine specialties such as oncology [41] and cardiology [42] and was persistent during the COVID-19 pandemic [43,44]. Ageism is significantly associated with poor health outcomes and quality of life [34,35]. Interventions to reduce ageism have been widely studied [45,46]. ChatGPT has been found to exhibit privacy, ethnicity, gender, and racial bias and entail legal risks [16-21]. For example, ChatGPT recommends fewer female than male ophthalmologists [47]. However, whether ChatGPT generates age-biased outputs has not been reported. This study aimed to demonstrate whether ChatGPT exhibits ageism by testing its geriatrics attitude using a validated University of California, Los Angeles (UCLA) geriatrics attitude instrument [48,49] and comparing its results with those obtained by medical students residents, and geriatrics medicine fellows from published articles

The second approach was to evaluate the geriatrics knowledge of ChatGPT. ChatGPT has passed the United States Medical Licensing Examination (USMLE) [50,51] and many other medicine exams [52-56], demonstrating its competence in medical knowledge. However, it has been less studied in geriatrics. For example, in one Geriatrics and AI in Spain project in Spain [30], ChatGPT was prompted with 10 questions about geriatric medicine. Compared to 130 physicians who answered the questionnaire, ChatGPT scored better on general and theoretical questions than on complex decisions and end-of-life situations, with the lowest scores for diagnosis and performing complex tests [30]. AI is likely to be incorporated into some areas of geriatric medicine, but it still presents significant limitations, mainly in complex medical decision-making [30]. This study was designed to demonstrate the geriatrics competency of ChatGPT by examining its performance on the validated UCLA geriatrics knowledge test [48,49,57] and comparing it with that of medical students, internal medicine residents, and geriatric medicine fellows from previously published articles in the literature.

The third approach was to evaluate ChatGPT’s knowledge of 2 geriatric syndromes and its clinical application to them (polypharmacy and falls). Multiple previous studies have shown that ChatGPT performs well on clinical vignette questions [58-64]. For example, ChatGPT achieved 71.7% accuracy overall on 36 clinical vignettes from the Merck Sharpe and Dohme clinical manuals and impressive accuracy in clinical decision-making [58]. In another study, GPT-4o was queried for diagnoses and management plans with 20 physician-written clinical vignettes in otolaryngology, showing high agreement with physicians [59]. In addition, ChatGPT demonstrated appreciable knowledge and interpretation skills in psychiatry through 100 clinical case vignettes [60]. In another study, 33 physicians across 17 specialties generated 284 medical questions. ChatGPT generated largely accurate information in response to diverse medical queries as judged by academic physician specialists, with improvement over time [61]. ChatGPT also offered accurate recommendations on managing hypertension based on clinical practice guidelines [62]. ChatGPT provided accurate recommendations for 8 out of 10 clinical scenarios in cardiology [63]. In another study, GPT-4o generated a good rehabilitation plan for a stroke case from a textbook [64]. Finally, several studies have reported the application of ChatGPT to predict common drug-drug interactions [65], managing polypharmacy [29], clinical pharmacy [66-68], and medical pharmacology as a self-learning tool [69]. Taken together, all previous studies suggest that ChatGPT could potentially be applied to complex geriatric syndrome vignettes with comprehensive clinical questions. This study was designed to demonstrate whether ChatGPT has solid geriatrics knowledge and can apply it to 2 common complex geriatric syndrome vignettes (polypharmacy and falls) by responding to comprehensive questions.

### Study Objectives

We designed these 3 distinct approaches to provide evidence of whether ChatGPT can be trusted to be potentially applied to geriatrics education and clinical practice as an assistive tool.

## Methods

This observational study used the validated UCLA geriatrics attitude instrument [48,49] and geriatrics knowledge test [57].

### The Geriatrics Attitude Instrument

The geriatrics attitude instrument comprises 16 questions (Multimedia Appendix 1) [48,49]. In total, 6 of these statements reflect positive attitudes toward aging, such as “Most old people are pleasant to be with” (question 1). A total of 10 statements reflect negative attitudes toward aging, such as “Treatment of chronically ill old patients is hopeless” (question 11). Responses are graded on a Likert scale from 1 (*strongly disagree*) to 5 (*strongly agree*). The mean total score of ChatGPT was calculated and compared to scores from medical students, internal medicine residents, and geriatric medicine fellows based on previously published studies in the literature. Subtotals for positive and negative attitudes toward aging were also calculated to compare them to those in previously published studies. For statements reflecting a positive attitude toward aging, higher scores indicated a more positive attitude toward aging. For statements reflecting a negative attitude toward aging, lower scores indicated a less biased attitude toward aging.

### The Geriatrics Knowledge Test

The validated UCLA geriatrics knowledge test contains 18 questions (Multimedia Appendix 2) [57]. In total, 8 questions are true-or-false statements, such as “Most older people are living in nursing homes” (question 4; false). A total of 10 questions are short clinical vignettes, such as “A 78-year-old nursing home resident with mild dementia associated with Alzheimer’s disease is best determined by their ability to understand treatment options” (question 9; correct answer: B).

To minimize unfounded guessing, the following scoring system was used: +1 for a correct answer, –1 for a wrong answer, and 0 for “don’t know.” Therefore, the total score ranged from −18 to +18 [57]. Correct answers were determined by the author based on the literature.

### ChatGPT Inputs and Outputs

GPT-3.5 was prompted to undertake the validated UCLA geriatrics attitude instrument and geriatrics knowledge test. Prompts were derived from original published papers [48,49,57]. Each prompt was repeated 4 times to evaluate the consistency and obtain an average response. In addition, ChatGPT was prompted to respond to 2 common geriatric syndrome vignettes (polypharmacy and falls). These 2 vignettes with questions were used in the author’s previously published geriatrics curricula [70,71] and are described fully in the following sections.

### The Polypharmacy Vignette

This vignette was used from a previously published workshop of prescribing and deprescribing [70]:

Ms. Smith, an 85-year-old white woman, has HTN, CAD, HFpEF, A-Fib, DM (II), HLD, hypothyroidism, gout, OA. On Warfarin 2.5 mg/d, furosemide 20 mg/d, Glyburide 5 mg/d, Spironolactone 50 mg/d, Amiodarone 300mg/d, Carvedilol 6.25 mg twice a day, Lisinopril 40 mg/d, Lipitor 80 mg/d, Levothyroxine 250 mg/d, Allopurinol 100 mg three times a day, acetaminophen 500 mg two tabs q6h for joint pain, and several supplements. Her pulse is 62 beat/min. Her BP is 120/70 (sit) and 98/64 (upright), weight 60 kg. No JVD, HR regularly, no murmur, few crackles on lung bases. Abdomen exam is benign, + pitting edema of ankles. Serum Cr is 1.2 mg/dL and Bun is 15 mg/dL. INR is 3.5. LDL is 50. albumin 4, Hb1ac is 6.1. TSH is 2. EF is 60%.

ChatGPT was prompted with the polypharmacy vignette following 2 steps. First, ChatGPT was prompted to answer 4 general questions on the appropriate use of medications to examine its geriatric pharmacology knowledge: (1) “Which of the commonly used 8 drugs are potentially inappropriate medication (PIM) and should be avoided in older adults?” (2) “What is PIM in older adults?” (3) “How to identify PIM in older adults?” (4) “What to do with PIM?” Second, ChatGPT was prompted to answer 4 additional specific questions related to the polypharmacy vignette to evaluate its application of geriatric pharmacology to a clinical vignette: (1) “Any drug-drug interactions for this patient?” (2) “Any drug is ineffective for this patient?” (3) “This patient was taking so many medications. Any way to reduce the number of her medications?” (4) “Any drug-disease interaction?”

### The Fall Vignette

This vignette was used from a previously published workshop of prescribing and deprescribing [71]:

An 82-year-old community-dwelling woman with PMH of multiple falls, right hip fracture, Dx of hypertension, coronary heart disease, knee arthritis, came to my clinic after she fell after her meal at home this morning. She felt pain of R head. She had a cane and walker at home but didn’t use that often. She thought she didn’t need it. She has taken care of her husband for several years. Her medication included Doxazosin (Cardura) and diltiazem (Cardizem), and acetaminophen as needed. I asked many questions related to her fall. Her vital signs were normal without orthostatic hypotension. Her cardiovascular and neurological examinations were benign. Her mini-mental status examination (MMSE) was 30/30. She had independent activities of daily living (ADL). Her geriatric depression scale (GDS) was 0/15. She passed timed up and go test. Because of her right black eye and pain of right head, I sent her to UVA to rule out orbital fracture. Please ask the following questions: List all potential fall risk factors for this patient What are three fall screening questions you need to ask for elderly patients in an outpatient setting based on 2010 fall prevention guideline you want to follow for this patient? Which one of three pathways in figure 1 from 2010 fall prevention guideline you want to follow for this patient? List all physical examinations that you think are important to perform for this patient. List your recommendations as many as you think that could help her to prevent her falling.

ChatGPT was prompted with the fall vignette following 3 steps. First, ChatGPT was prompted to answer 1 general question on fall risks in older adults. Second, ChatGPT was prompted to answer five pretest questions on fall prevention in older adults: (1) “When an older person falls, should you ask the following questions (one or more or none)? the circumstances of fall, mental status, medication changes, vision change, drinking”; (2) “Which of the following illnesses is a fall risk (one or more or none)? Pneumonia, Parkinson’s disease, Diabetes mellitus, Dementia, Orthostatic hypotension”; (3) “Which of the following medications are not associated with an increased fall risk (one or more)? Levothyroxine, Diazepam, Oxycodone, Amitriptyline”; (4) “Which ones of the following are true (one or more or none)? An elderly person may become so fearful of falling that they restrict his or her mobility Most falls are associated with significant injuries Falls is a leading cause of accidental death in older population About 5% community-dwelling elderly person falls each year Survivors of fall-related hip fracture are rarely institutionalized Exercise improves function and reduces fall risk and injurious falls”; and (5) “Which of the following is NOT an environmental fall risk? (only pick one answer) Throw rug, freshly waxed kitchen floor, Grab bars, Electrical cord lying on the floor.” Finally, ChatGPT was prompted to answer 5 additional questions to examine the application of fall prevention knowledge to the fall vignette: (1) “List all potential fall risk factors for this patient”; (2) “What are three fall screening questions you need to ask for elderly patients in an outpatient setting based on 2010 fall prevention guideline you want to follow for this patient?”; (3) “Which one of three pathways in figure 1 from 2010 fall prevention guideline you want to follow for this patient?”; (4) “List all physical examinations that you think they are important to perform for this patient”; and (5) “List your recommendations as many as you think that could help her to prevent her falling.”

### Evaluation of ChatGPT Outputs

ChatGPT outputs were collected and analyzed by the author. The author judged the correctness of the ChatGPT outputs on the polypharmacy and fall vignette questions based on evidence from clinical practice guidelines, systematic reviews, and other types of evidence in the literature. The descriptive analysis was performed using SPSS (version 28; IBM Corp).

In summary, we designed these 3 distinct approaches to provide evidence on whether we can trust ChatGPT to be potentially applied to geriatrics education and clinical practice as an assisting tool.

### Ethical Considerations

This study was not human participant related, including the secondary data analysis. No informed consent was needed.

## Results

### Geriatrics Attitude

The total geriatrics attitude score of ChatGPT was significantly lower than that of trainees (medical students, internal medicine residents, and geriatric medicine fellows) from validation [49,57] and follow-up studies [48] (Multimedia Appendix 3). However, the mean subscore on positive geriatrics attitude of ChatGPT was higher than that of the trainees (medical students, internal medicine residents, and neurologists; Multimedia Appendix 4), where a higher score is better. The higher subscore on positive geriatrics attitude indicates a better attitude toward aging. Conversely, the mean subscore on negative geriatrics attitude of ChatGPT was lower than that of the trainees and neurologists, where lower subscores are better (Multimedia Appendix 4). The lower subscore on negative geriatrics attitude indicates a less age-biased attitude toward aging. Individual responses to 14 geriatrics attitude statements by ChatGPT, trainees, and neurologists are shown in Multimedia Appendix 4. The subscores of the comparison group in Multimedia Appendix 4 were based on previously published studies [72-74]. Notably, responses to statements 15 and 16 were rarely reported in previously published studies (Multimedia Appendix 4).

Of the 14 geriatrics attitude statements (Multimedia Appendix 4), 5 (36%) were positive, and 9 (64%) were negative. Regarding the positive geriatrics attitude statements, ChatGPT had 1 lower positive geriatric attitude response (indicating a less positive geriatrics attitude) and 5 similar or better positive geriatric attitude responses compared to medical students and residents (indicating a similar or better positive geriatrics attitude). Regarding the negative geriatrics attitude statements, ChatGPT had 1 similar negative geriatric attitude response and 8 better negative geriatrics attitude responses than medical students and residents, indicating a less negative geriatrics attitude.

The response from ChatGPT to the first prompt of the geriatrics attitude questions did not follow the Likert-scale format (1-5; 1=*strongly disagree*; 5=*strongly agree*; Multimedia Appendices 3 and 4) as ChatGPT provided only comments. The response to the third prompt included both the Likert-scale format (1-5; 1=*strongly disagree*; 5=*strongly agree*) and comments (Multimedia Appendix 4).

### Geriatrics Knowledge

The total score of ChatGPT (mean 14.25) was significantly higher than the scores from all trainees (medical students, residents, and internal medicine fellows) in the validation studies [57] (mean 11.3; Multimedia Appendix 3) and was also significantly higher than that of first-year (mean 9.9) and second-year (mean 9.5) medical students (Multimedia Appendix 3) in the follow-up studies [48]. It was slightly higher than the scores of third-year medical students (mean 13.6) and internal medicine interns (mean 13.2), slightly lower than the scores of second- and third-year internal medicine residents (mean 14.7 and 14.9, respectively), and significantly lower than the scores of geriatric medicine fellows (mean 17.5) in the follow-up studies [48]. ChatGPT’s performance on geriatrics knowledge in the first prompt was lower than that on the following 3 repeat prompts. For the first prompt, ChatGPT provided rationales without selecting options (Multimedia Appendix 4). For the third prompt, ChatGPT selected options and provided rationales for its choices (Multimedia Appendix 4). For the second and fourth prompts, ChatGPT select options without providing rationales (Multimedia Appendix 4).

To make it easy for the reader to replicate the results of this study, Multimedia Appendix 5 shows a few examples of prompts on geriatrics attitude and knowledge test questions and outputs by ChatGPT (all original outputs of ChatGPT are available upon request).

### The Polypharmacy Vignette

Overall, ChatGPT performed well on the polypharmacy vignette involving a woman aged 85 years (Multimedia Appendix 6). ChatGPT provided appropriate responses to 4 general drug therapy questions and moderate responses to 4 specific drug therapy questions based on the vignette. ChatGPT correctly identified 5 drug-drug interactions and suggested deprescribing. However, it missed an ineffective medication (a supplement), 1 drug-drug interaction, and 3 drug-disease interactions (Multimedia Appendix 6). Despite the patient not having lung disease, ChatGPT provided 2 irrelevant drug-disease interactions specific to the vignette (ChatGPT hallucination; Multimedia Appendix 6).

### The Fall Vignette

ChatGPT performed well on the fall vignette involving a woman aged 82 years (Multimedia Appendix 7). ChatGPT correctly summarized 10 common fall risks in older adults and correctly answered 3 out of 5 pretest questions. In the remaining 2 pretest questions, ChatGPT missed a few correct responses. In the fall vignette, ChatGPT provided appropriate responses to all 4 prompts (Multimedia Appendix 7). ChatGPT recognized most fall risk factors and responded perfectly to fall screening questions following the latest Centers for Disease Control and Prevention (CDC) fall guidelines even though the prompt specified the 2010 fall prevention guidelines. ChatGPT accurately followed the 3 pathways from the 2010 fall guidelines. It missed a few physical examinations. ChatGPT provided almost all recommended fall prevention strategies, with only a few omissions.

## Discussion

### Principal Findings

With the increasing use of ChatGPT in medical education [3-7] and clinical practice [5-12], the aim of this study was to evaluate whether we can trust ChatGPT to be used in geriatrics. The major findings of this study demonstrated that ChatGPT had less age-biased output than the trainees, outperformed the trainees on the validated UCLA geriatrics knowledge test, and reasonably applied geriatrics knowledge to 2 common geriatric syndrome vignettes (polypharmacy and falls). The preliminary findings of this study are promising and demonstrate that ChatGPT could be trusted and used as an assistant tool in geriatrics practice and education via 3 approaches, which are fully discussed in the following paragraphs.

The first approach was to evaluate ChatGPT’s performance on a validated UCLA geriatrics attitude instrument [48,49]. To the best of our knowledge, this is the first study to demonstrate that ChatGPT is less likely to generate age-biased outputs, which contrasts with a few ethical and other concerns regarding ChatGPT [16-21]. This finding is significant because ageism is prevalent and associated with multiple poor outcomes, including health outcomes [34,35]. Various instruments exist to measure geriatrics attitude to assess ageism [34,35], with the UCLA geriatrics attitude instrument [48,49] being one of these validated tools [75], which we used in this study [48,49]. The UCLA geriatrics attitude instrument includes 16 items [48,49], 6 of which refer to a positive geriatrics attitude (eg, “most old people are pleasant to be with”) where a higher score is better, and 10 of which refer to a negative geriatrics attitude where a lower score is better. Previous studies using the UCLA geriatrics attitude instrument have primarily used the total score on the geriatrics attitude scales without calculating and reporting the mean subscores on positive and negative geriatrics attitudes [48,49], which makes it difficult to interpret the significance of geriatrics attitude. However, a few studies have reported individual responses to the UCLA geriatrics attitude instrument, including positive and negative geriatrics attitude [48,49], for which we were able to calculate the subscores for comparison. We recommend that the subscores of the UCLA geriatrics attitude instrument should be reported to assess positive and negative geriatrics attitude. Overall, ChatGPT’s individual responses to the UCLA geriatrics attitude statements were better than those of medical students and residents, with higher scores for positive attitude and lower scores for negative attitude (Multimedia Appendix 4), suggesting that ChatGPT has less age-biased outputs and could be trusted from an ethical perspective. This study suggests that ChatGPT is better than the news media and humans in providing less age-biased information [39,40]. However, a reliable and valid instrument with which to quantify modern medical student attitudes toward older people has not yet been developed. An adaptation of the Aging Semantic Differential scale for contemporary use has been recommended [76]. This study can be repeated using the Aging Semantic Differential scale to see whether ChatGPT still generates less age-biased outputs in the future.

The second approach was to evaluate ChatGPT’s geriatrics knowledge. This study is the first to assess ChatGPT’s performance on a validated UCLA geriatrics knowledge test [49,57]. ChatGPT has a substantial knowledge base and has been reported to pass the USMLE [50,51] and perform well on many other examinations [52-56]. Previous studies have suggested that ChatGPT could significantly impact medical education [3-7] and clinical practice, including clinical decision-making [5-12]. For example, a recent systematic review and meta-analysis on the performance of ChatGPT in medical examinations showed that ChatGPT has been evaluated in multiple specialties, including plastic surgery, ophthalmology, neurosurgery, orthopedics, diabetes, gastroenterology, radiology, cardiology, dermatology, and anesthesia [61]. It can be reasonably believed that ChatGPT has good geriatrics knowledge. This study supported this belief and demonstrated that ChatGPT performed better than the average trainee in the validation studies (mean score 14.7 vs 11.3) and follow-up studies (mean score 14 vs 13.3; Multimedia Appendix 3). ChatGPT’s performance was better than that of medical students and first-year internal medicine residents (PGY 1 in Multimedia Appendix 3), comparable to that of second- and third-year residents (PGY 2 and PGY 3 in Multimedia Appendix 3), but lower than that of geriatric medicine fellows. This suggests that ChatGPT could be a valuable supplemental resource for health professional trainees, health care providers, and laypeople. Given that GPT-4o has more parameters, it is reasonable to believe that GPT-4o could perform better on the geriatrics knowledge test. The UCLA geriatrics knowledge test only has 18 questions [57] and much less questions than the USMLE tested in previous studies [50,51]. Future studies should evaluate GPT-4o’s performance on more multiple-choice questions from geriatrics board examinations to confirm the findings of this study before adopting ChatGPT for geriatrics education and clinical practice.

The third approach was to evaluate ChatGPT’s responses to 2 common geriatric syndrome vignettes—polypharmacy and falls in older adults—from the author’s previously published geriatrics curricula [70,71]. This study demonstrated that ChatGPT performed well on these 2 geriatric syndrome vignettes (Multimedia Appendices 6 and 7), which was promising and will be fully discussed in the following paragraphs.

For the polypharmacy vignette, ChatGPT was able to provide systematic recommendations for polypharmacy in older adults, which has not been investigated before. In this study, ChatGPT provided appropriate general principles for prescribing and deprescribing; identified PIMs; and suggested resources and criteria such as the Beers criteria [77], the Screening Tool of Older Persons’ Prescriptions [75], the Drug Burden Index [78], and the Medication Appropriateness Index [79] (Multimedia Appendix 6). In addition, ChatGPT was able to provide 5 appropriate ways to manage PIMs in older adults (Multimedia Appendix 6) that are consistent with the evidence from geriatrics practice guidelines and prescribing principles [75,77-80]. In particular, this study demonstrated that ChatGPT can identify all 8 exemplary PIMs (Multimedia Appendix 6). These medications should be avoided in older adults [75,77-80]. ChatGPT was able to identify 4 drug-disease interactions but missed 3 drug-disease interactions in the polypharmacy vignette (Multimedia Appendix 6). This suggests room for improvement in training ChatGPT further. ChatGPT also identified drug-drug interactions accurately in the polypharmacy vignette (Multimedia Appendix 6), which was different from a previous study in which ChatGPT identified 39 out of 40 drug-drug interactions when it was given 40 pairs of drugs [65]. Therefore, the question is what the implications are of what ChatGPT can do for polypharmacy. Previous studies have shown that within the last 10 years medical students, postgraduate residents, primary care providers, and pharmacists were unaware of PIMs and the standard guidelines for older adults, such as the Beers criteria [81-84]. This study has shown that ChatGPT is more knowledgeable than humans. Both geriatricians and general internists still prescribed 7.2% to 8.7% of PIMs, respectively [84].

The persistent prevalence of PIMs and polypharmacy in older adults was well-reported [31,32]. What ChatGPT demonstrated its good knowledge and ability to do with polypharmacy in this study suggests that ChatGPT has great potential to be used by trainees and providers as an assistant tool. One suggested example will be to include an older patient’s medical history and a list of all their medications in the ChatGPT prompt used in this study for medication review and to generate ChatGPT outputs which can be reviewed by providers at Clinic or other clinical settings. This will help nurses, other providers, and trainees at the point of care. It could help laypeople self-check PIMs. It could also help health professional trainees in the self-study of geriatric pharmacology.

Falling is another significant US and global problem [85,86] and one of the common geriatric syndromes in older adults [87]. ChatGPT has not been tested to provide systematic fall prevention recommendations for an older adult. This study demonstrated for the first time that ChatGPT has good knowledge of fall prevention as it performed well on pretests and provided a comprehensive summary of 10 common fall risks in older adults and specific recommendations in the fall vignette (Multimedia Appendix 7).

The 2024 US Preventive Services Task Force fall prevention guidelines listed several fall risks, including age; history of falls; cognitive and sensory deficits; presence of acute and chronic medical conditions; certain medications; environmental or occupational hazards; home or neighborhood features and alcohol or drug use; and impairments in mobility, gait, and balance [87]. The 2023 CDC Stopping Elderly Accidents, Deaths, and Injuries also has a long list of fall risks [88]. In addition, a systematic review and meta-analysis showed evidence-based fall risk factors among the aging population [89]. This study showed that ChatGPT identified most of fall risks for the fall vignette (Multimedia Appendix 7) consistent with the aforementioned fall prevention guidelines [87,88] and systematic review [89] except for missing risky behaviors such as standing on a chair instead of a step stool and loss of sensation in the feet.

This study also used 5 pretests to further evaluate ChatGPT’s knowledge of fall prevention (Multimedia Appendix 7). ChatGPT generated responses consistent with the guidelines [88,89] and a systematic review [90]. In pretest 1, ChatGPT provided 5 good recommendations with reasoning for taking the medical history of an older adult. In pretest 2, ChatGPT correctly identified several medical conditions as fall risks, including Parkinson disease, dementia, and orthostatic hypotension but missing pneumonia and diabetes mellitus. This indicates that ChatGPT has some limitations. In pretest 3, ChatGPT correctly identified diazepam, oxycodone, and amitriptyline as fall risks [75,77-79] and levothyroxine as not a fall risk. In pretest 4, ChatGPT underestimated the prevalence of falls in community-dwelling older adults (5%) but correctly answered most other true and false questions about fall risks and consequences. In pretest 5, ChatGPT correctly answered all questions about environmental fall risks and identified grab bars as not a risk. Overall, ChatGPT performed very well on 5 pretests with a few mistakes, indicating good knowledge of fall prevention for older adults.

To further test ChatGPT’s clinical application to individual patients, the fall vignette was used to prompt ChatGPT. ChatGPT correctly identified 6 common fall risk factors except for potential home environment factors, consistent with the evidence from fall prevention guidelines [88,89] and a systematic review [90]. ChatGPT impressively recognized older caregiver burden, hypertension, and coronary heart diseases as fall risks, also consistent with the evidence from fall prevention guidelines [88,89] and a systematic review [90]. In response to fall screening questions, ChatGPT was correct if the 2023 CDC guidelines were used [89] but incorrect if the 2010 American Geriatrics Society guidelines were used [91]. This study indicates that ChatGPT can use the latest guidelines, although specific prompts might be unclear to ChatGPT. Regarding physical examinations relevant to the fall vignette, ChatGPT missed several but recommended multiple appropriate physical examinations [89,91], indicating a reasonably good performance. In the final prompt on fall prevention recommendations for the fall vignette, ChatGPT provided 6 appropriate fall prevention recommendations, including home environment–related prevention despite previously missing the home environment as a fall risk [88-91]. It was notable that the home environment was not on the list of fall risks in ChatGPT’s response to the first prompt. However, ChatGPT correctly answered pretest 5 (home environment fall risk) and recommended home environment–related prevention for the patient in the fall vignette (Multimedia Appendix 7). This suggests some discordance between the fall assessment and action plans by ChatGPT, indicating a possibly different thinking and decision process within ChatGPT.

The crucial question is whether ChatGPT can be helpful and necessary in screening for fall risks and providing fall prevention recommendations to older adults in daily practice. Providers need knowledge and willingness to screen for fall risks in older adults, but multiple studies have shown that providers often lack fall prevention knowledge and are unwilling to screen for fall risks [92-96]. For example, one study showed that emergency providers lacked knowledge on which patients to be screened and were unwilling to spend more than a few minutes on screening for fall intervention [95]. Another study revealed that only 14% of providers at accountable care organizations were aware of the CDC’s fall risk assessment algorithm (Stopping Elderly Accidents, Deaths, and Injuries) [93]. Furthermore, 43% of primary care providers did not agree that they had the expertise or time to perform fall risk assessments, and only a small percentage billed for fall risk screening despite being aware of Medicare reimbursement [96]. In addition, general practitioners were often unaware of their frail patients’ fall history or fear of falling, with most patients not receiving fall prevention care. Less than 40% of providers asked most or all their older patients if they had fallen in the previous 12 months, and less than a quarter referred their patients to physical therapists for balance or gait training [92]. A recent CDC report showed some improvement, but gaps remain. Only 20% of providers were aware of any injury prevention resources, and a higher percentage screened for fall risk when older adults presented with specific concerns [94].

Given the high prevalence of fall among older adults and poor knowledge of fall prevention among providers, this study demonstrated that ChatGPT has better knowledge and ability to apply the fall prevention guidelines than many providers, suggesting that it can assist providers in assessing fall risks and preventing falls in older adults. For instance, entering an older patient’s history into ChatGPT and reviewing the outputs generated can help providers identify patients with fall risk at the point of care. ChatGPT can also be used as an assistant tool to help health professional trainees with self-study.

### Limitations and Quality Assessment of This Study

There are serval limitations to this study, which will be discussed in this section. ChatGPT’s responses are based on the data that it was trained on, which may not include the most current geriatrics research, best practices, or region-specific guidelines, potentially limiting the trustworthiness of its advice. This study did not account for the quality of the information sources that ChatGPT draws from as ChatGPT cannot differentiate between authoritative and nonauthoritative content in real time like all other ChatGPT-based research. Despite GPT-4o having more data for training without releasing its data sources, this will not solve this basic problem. This study indicates that applying ChatGPT to geriatrics practice and education should be cautious and it should be used as an assistant tool only. ChatGPT will never or is unlikely to replace clinicians. As this study evaluated ChatGPT based on geriatric syndrome vignettes, it does not fully account for the complexities and nuances of real-world clinical decision-making and patient interactions, where additional context, history, and human judgment play critical roles. In addition, ChatGPT’s responses might be overly generalized as it lacks the ability to perform individualized assessments or make nuanced clinical decisions based on patient-specific data. This is similar to vignette-based simulation education. This indicates that ChatGPT will not make a decision for someone but could assist with the decision-making. The effectiveness of ChatGPT’s responses varies significantly depending on how well the user phrases the questions or provides necessary information. The input provided in this study was based on the author’s geriatrics practice experience for >20 years, which might not mimic the way in which other clinicians would interact with the tool. Therefore, the results of this study may not be generalizable. Prompt engineering is developing to help improve the quality of the input. However, a standardized prompts among different providers can be very challenging. The author suggests practicing prompts to improve human-ChatGPT interaction and obtain reliable outputs from ChatGPT.

ChatGPT lacks clinical experience and the ability to engage in human clinical judgment, whereas it can process vast amounts of medical information. This may limit its application in critical decision-making scenarios or nuanced treatment planning for complex geriatrics cases. Providers must use their clinical judgment integrated with clinical circumstances and not fully depend on the outputs of ChatGPT.

ChatGPT might face ethical and legal consequences. ChatGPT or AI hallucinations could cause ethical and legal risks. After the release of GPT-4o, ChatGPT often says, “I am AI and not clinician and cannot make decisions for you.” It seems that ChatGPT developers recognize the potential legal and ethical consequences and protect ChatGPT.

Human-AI interaction is complex. Providers should be cautious in interpreting ChatGPT outputs. ChatGPT outputs should be interpreting with evidence-based clinical guidelines, systematic reviews, and randomized controlled trials shown in the Comments from the author columns in Multimedia Appendices 6 and 7. Whether the ChatGPT outputs are appropriate in this study should be judged and verified by a group of geriatricians

In addition, this study has several other limitations. This study used GPT-3.5. GPT-4o might produce better results. Comparisons between ChatGPT and human performance on geriatrics attitude and knowledge were based on previously reported results in the literature many years ago. A new study assessing geriatrics attitude and knowledge among current medical students, residents, and geriatric medicine fellows is needed to confirm the findings of this study. ChatGPT produced some errors, including ChatGPT hallucinations such as unrelated drug-disease interactions in Multimedia Appendix 6, consistent with previous reports [97,98]. We should be aware that ChatGPT can make mistakes, just like humans. ChatGPT should not be used alone in geriatrics practice and education at this point. ChatGPT needs to be pretrained specifically for medical education and clinical practice.

Like any other research, systematic appraisal of the current ChatGPT research is needed. A preliminary checklist, Model, Evaluation, Timing, Range and Randomization, Individual Factors, Counts, and Specificity of Prompts and Language (METRICS), to standardize the design and reporting of studies on generative AI–based models in health care education and practice has been recently developed [99]. The current study was started and completed before its development. However, it is worthwhile to use METRICS to evaluate the quality of this study. The following seven domains of METRICS will be used:

1. Model—ChatGPT was described and used.
2. Evaluation—both subjective and objective evaluation were described and used.
3. Timing—the study date was documented, but the duration was not documented; transparency—the geriatrics attitude instrument and knowledge test, the vignettes, and prompts were well described, and the outputs were exactly presented.
4. Range—the topics were well described, including geriatrics attitude and knowledge; randomization was not used.
5. Individual—there was subjective involvement with ChatGPT, such as judging the correctness of its outputs. This study was conducted by a single investigator.
6. Count—a total of 4 prompts were used. This study did not have a sample size.
7. Specificity of the prompts or language—for geriatrics attitude and knowledge, the prompts were from previously published studies. It is unclear whether they are appropriate for ChatGPT. For the 2 vignettes on polypharmacy and fall, the prompts were from previously published curricula. It is also unclear whether they are appropriate for ChatGPT.

Overall, the quality of this study is reasonably good based on METRICS. METRICS should be used in the reporting of any AI-based research in all journals.

In summary, this study took 3 distinct approaches to demonstrate the trustworthiness of ChatGPT to be used as an assistant tool in geriatrics practice and education. The implications of this preliminary study for geriatrics practice and education are further discussed in the following section.

### Implications for Geriatrics Practice and Education

This study showed that ChatGPT outputs are not age-biased using the validated geriatrics attitude test and that ChatGPT performed well on the validated geriatrics knowledge test and on applied geriatrics knowledge tests (2 cases of common geriatric syndromes). These findings suggest that ChatGPT could potentially help geriatrics practice and education as an assistant tool in numerous ways. It is very important to know that ChatGPT is algorithm based. Clinical practice often uses algorithm- and pathway. Therefore, the underlying rationale for ChatGPT and clinical practice is similar. However, ChatGPT is not intended to replace physicians and other providers.

ChatGPT can be used as an assistant geriatrics tool and tutor to support self-learning and immediate feedback and self-assessment. For example, when trainees are studying geriatric syndromes, they can obtain responses on geriatric syndromes from ChatGPT. Therefore, ChatGPT could be incorporated to supplement the existing program in geriatrics education and support continuing medical education. ChatGPT can be potentially to be integrated to clinical reasoning and decision in a timely fashion and potentially improve the point of care in all clinical settings. For example, ChatGPT can help identify individual older patients who might have fall risks and drug-drug interactions.

ChatGPT can be used as an assistant or autonomous clinical tool to alleviate the geriatrics workforce crisis. For example, a nongeriatrician health provider could look for answers to clinical geriatrics questions, such as recommendations for preventing falls and reducing polypharmacy in older patients, from ChatGPT and help answer their patients’ questions in a timely fashion. This is particularly helpful for rural practice where geriatrics consults are rare or nonexistent. This will improve physicians’ and other providers’ efficiency and accuracy in the care of older patients and provider-patient interactions.

It is expected that ChatGPT can help with post-visit summaries by providing patient education materials, such as home safety and environment modification for fall prevention. ChatGPT can also draft Clinic letters such as providing lab results and their interpretation to the patient in the Clinic letter.

ChatGPT can help older patients with self-screening or initial self-assessment when they are sitting in a clinic waiting room. For example, they can input their medical history into ChatGPT such as age, physical function medications, and chronic conditions to ask whether they are at higher risk of fall.

Importantly, GPT-3.5 is free to use and can be cost-saving for providers and the health care system.

Finally, with more data training, ChatGPT will be more reliable and helpful in geriatrics practice and education.

### Comparison With Previous Work

To the best of our knowledge, health professional trainees and providers but not ChatGPT have been assessed for geriatrics attitude [48,49]. ChatGPT has been assessed for geriatrics knowledge [28-30]. For example, ChatGPT was asked to answer 10 geriatrics questions [30]. The correctness of the outputs by ChatGPT were rated by a group of geriatricians [30]. In contrast, in this study, the correctness of the outputs by ChatGPT was determined by the author only, which could lead to errors. In another study, ChatGPT was prompted with 1 simple question [28]. The questions in the aforementioned studies were not validated. However, this study used validated UCLA geriatrics knowledge tests [57,72-74]. Several previous studies have demonstrated that ChatGPT has geriatric pharmacology knowledge [29,65-69]. For example, ChatGPT could identify drug-drug interactions from 40 drug-drug pairs [65]. In another study, ChatGPT could manage polypharmacy and make decisions on deprescribing 2 to 3 inappropriate medications based on a vignette [29]. Another study compared the answers from ChatGPT and clinical pharmacists to real clinical cases and clinical competency assessments [68]. However, these studies are different from this study, which used a systemic approach to polypharmacy (Multimedia Appendix 6). To the best of our knowledge, ChatGPT has not approached falls in older adults.

### Future Directions

This study was a pilot with both promising findings and limitations. With the fast-growing use of ChatGPT and the new version of GPT-4o being released, the author suggests the following research directions.

A larger study using ChatGPT is needed to extend and confirm the findings of this study before adopting ChatGPT for geriatrics education and clinical practice. For example, more geriatrics knowledge questions and vignettes should be examined using GPT-4o. For example, the performance of ChatGPT on geriatrics certification exams should be tested.

Coinvestigators are needed to reduce biases. In particular, the correctness of the ChatGPT outputs should be evaluated and judged by a group of experts in geriatrics and integrated with the latest evidence, such as clinical practice guidelines, systematic reviews, and randomized controlled trials. For example, the ChatGPT outputs to a prompt should be rated from *strongly agree* to *strongly disagree* by a group of geriatrics experts in addition to determining their consistency with evidence-based clinical practice guidelines

Those who conduct ChatGPT research should receive training on prompt engineering. The author is doing this and feels that it is beneficial. This could reduce the variation in prompts.

The reliability of ChatGPT performance needs to be tested using different prompts to improve generalizability. The outcomes of the application of ChatGPT to geriatrics practice and education need to be further investigated.

### Conclusions

This study suggests that ChatGPT could be a valuable assistant tool in geriatrics education and practice, helping health professional trainees and providers combat ageism, supplement geriatrics knowledge, and address common geriatric syndromes such as polypharmacy and falls. This study demonstrated that we could trust ChatGPT to be used in geriatrics practice and education by using 3 distinct approaches. One strength of this study is that Multimedia Appendices 5-7 provide the details of prompts to ChatGPT and the outputs generated by ChatGPT, which allows readers to apply a similar approach to their geriatrics education and practice studies. The findings of this study are promising but need more investigation before ChatGPT is widely adopted in geriatrics practice and education.

## Appendix

Multimedia Appendix 1 Geriatrics attitudes tests.

Multimedia Appendix 2 University of California, Los Angeles, geriatrics knowledge test (questions 1-18).

Multimedia Appendix 3 Comparison of geriatrics attitude and knowledge test performance between ChatGPT and trainees.

Multimedia Appendix 4 Comparison of performance on the geriatrics attitude subscales by ChatGPT, trainees, and neurologists.

Multimedia Appendix 5 Examples of ChatGPT outputs on geriatrics attitude and knowledge questions.

Multimedia Appendix 6 ChatGPT output on the polypharmacy vignette about a woman aged 85 years.

Multimedia Appendix 7 Fall risk identification and management in a woman aged 82 years.

## Abbreviations

AI: artificial intelligence
CDC: Centers for Disease Control and Prevention
METRICS: Model, Evaluation, Timing, Range and Randomization, Individual Factors, Counts, and Specificity of Prompts and Language
PIM: potentially inappropriate medication
UCLA: University of California, Los Angeles
USMLE: United States Medical Licensing Examination

## Data Availability

All data generated or analyzed during this study including all ChatGPT prompts and outputs are included in this published article and its supplementary information files on the journal website.
