# Supplementary material for: ChatGPT’s Attitude, Knowledge, and Clinical Application in Geriatrics Practice and Education: Exploratory Observational Study
Source: JMIR Form Res. 2025 Jan 3;9:e63494. doi: 10.2196/63494 (PMC11742095; doi:10.2196/63494)
Supplement: Multimedia Appendix 1 [file formative_v9i1e63494_app1.docx]

**Appendix 1. Geriatrics attitudes tests**

**DIRECTIONS: Please use the scale to indicate the degree to which you agree or disagree with each statement. There are no right or wrong answers. The best response is the one that truly reflects your personal opinion. Findings of this study will be reported only on a group basis with no individual names identified. “Old people” and “elderly patients” mentioned in the questions refer to persons aged 65 or older.**

|  | **Strongly Disagree** | **Somewhat Disagree** | **Neutral** | **Somewhat Agree** | **Strongly Agree** |
| --- | --- | --- | --- | --- | --- |
| **1. Most old people are pleasant to be with.** | **1** | **2** | **3** | **4** | **5** |
| **2. The federal government should reallocate money from Medicare to research on AIDS or pediatric diseases.** | **1** | **2** | **3** | **4** | **5** |
| **3. If I have the choice, I would rather see younger patients than elderly ones.** | **1** | **2** | **3** | **4** | **5** |
| **4. It is society's responsibility to provide care for its elderly persons.** | **1** | **2** | **3** | **4** | **5** |
| **5. Medical care for old people uses up too much human and material resources.** | **1** | **2** | **3** | **4** | **5** |
| **6. As people grow older, they become less organized and more confused.** | **1** | **2** | **3** | **4** | **5** |
| **7. Elderly patients tend to be more appreciative of the medical care I provide than are younger patients.** | **1** | **2** | **3** | **4** | **5** |
| **8. Taking a medical history from elderly patients is frequently an ordeal.** | **1** | **2** | **3** | **4** | **5** |
| **9. I tend to pay more attention and have more sympathy towards my elderly patients than my younger patients.** | **1** | **2** | **3** | **4** | **5** |
| **10. Old people in general do not contribute much to society.** | **1** | **2** | **3** | **4** | **5** |
| **11. Treatment of chronically ill old patients is hopeless.** | **1** | **2** | **3** | **4** | **5** |
| **12. Old persons don't contribute their fair share towards paying for their health care.** | **1** | **2** | **3** | **4** | **5** |
| **13. In general, old people act too slow for modern society.** | **1** | **2** | **3** | **4** | **5** |
| **14. It is interesting listening to old peoples’ accounts of their experience.**  **15. I feel comfortable working with elderly adults**  **16. Most elders feel uncomfortable discussing the issue of death and dying with their physicians** | **1**  **1**  **1** | **2**  **2**  **2** | **3**  **3**  **3** | **4**  **4**  **4** | **5**  **5**  **5** |

**References [48,49]**
