# Supplementary material for: ChatGPT’s Attitude, Knowledge, and Clinical Application in Geriatrics Practice and Education: Exploratory Observational Study
Source: JMIR Form Res. 2025 Jan 3;9:e63494. doi: 10.2196/63494 (PMC11742095; doi:10.2196/63494)
Supplement: Multimedia Appendix 2 [file formative_v9i1e63494_app2.docx]

Appendix 2: UCLA Geriatrics Knowledge Tests (1-18)

| The following items are designed to measure the progressive growth of your knowledge in geriatric medicine. You are therefore not expected to know all the answers until you are ready to graduate from medical school. | | | | |
| --- | --- | --- | --- | --- |
| However, to minimize unfounded guessing, we are using a slightly unusual scoring system. The survey will be scored with a +1 for a correct answer, a −1 for an incorrect answer, and a 0 for the don't know choice. Please answer “Don't know” for those items that you truly have no idea of the right answer. | | | | |
| **DIRECTIONS:** PLEASE CIRCLE “T” FOR “TRUE,”“F” FOR “FALSE” OR “?” FOR “DON'T KNOW” ON THE FOLLOWING ITEMS. | | | | |
| T | F | ? | 1. | Memory loss is a normal part of aging. |
| T | F | ? | 2. | A person's height tends to decline in old age. |
| T | F | ? | 3. | People 65 years of age and older make up about 20 percent of the current U.S. population. |
| T | F | ? | 4. | Most older people are living in nursing homes. |
| T | F | ? | 5. | Social Security benefits automatically increase with inflation. |
| T | F | ? | 6. | Most older drivers are quite capable of safely operating a motor vehicle. |
| T | F | ? | 7. | Most older adults consider their health to be good. |
| T | F | ? | 8. | Older females exhibit better health care practices than older males. |

Source: Adapted from Palmore, E. The Facts on Aging Quiz, 2nd Ed. New York: Springer, 1998.

DIRECTIONS: EACH OF THE QUESTIONS OR INCOMPLETE STATEMENTS BELOW IS FOLLOWED BY SUGGESTED ANSWERS OR COMPLETIONS. SELECT THE ONE THAT IS BEST IN EACH CASE. USE THE “DON'T KNOW” CHOICE WHEN YOU HAVE NO BASIS FOR MAKING AN EDUCATED GUESS.

9. A 78-year-old nursing home resident has mild dementia associated with Alzheimer's disease. She is disoriented to time and place but knows family members and regular nurse aides by name. This patient's capacity to make decisions regarding her health care is best determined by:

(A) Mental status test

(B) Her ability to understand treatment options

(C) Probate court decision

(D) Psychiatric examination

(E) Don't know

10. Which of the following instruments is most appropriate to assess physical functional abilities in a 75-year-old nursing home resident?

(A) Activities of Daily Living Scale

(B) Face/Hand Test

(C) Instrumental Activities of Daily Living Scale

(D) Zung Self-Rating Depression Scale

(E) Don't know

11. An 82-year-old nursing home resident has had a 7-lb weight loss over the past two months. She had a stroke 7 years ago that resulted in aphasia. She is bed bound and needs assistance when eating. The staff reports that she often clamps her teeth and refuses to eat. Physical examination shows no abnormalities. The most appropriate next step in management is:

(A) Observation

(B) Contact the family

(C) Begin nasogastric tube feeding

(D) Admit to the hospital

(E) Perform percutaneous endoscopic gastrostomy

(F) Don't know

12. During a home visit on an 85-year-old woman with a history of frequent falls, you perform a home safety evaluation. Your recommendations for environmental modifications might include each of following EXCEPT:

(A) Remove throw rugs

(B) Purchase a cordless phone

(C) Purchase low-lying furniture

(D) Install handrails on stairs

(E) Use rubber mats in the bathtub

(F) Don't know

13. The most common community-acquired infection that results in hospitalization in older persons is:

(A) Infected pressure ulcers

(B) Influenza

(C) Pneumonia

(D) Tuberculosis

(E) Urosepsis

(F) Don't know

14. A 66-year-old female acknowledges on review of systems that she often looses small amounts of urine when she coughs, sneezes, or does low-impact aerobics. She has considered quitting aerobics classes because she is so embarrassed. The most likely diagnosis is:

(A) Urge incontinence

(B) Overflow incontinence

(C) Stress urinary incontinence

(D) None of the above

(E) Don't know

15. A 70-year-old man with diabetes mellitus and emphysema comes to your office in December for influenza vaccination. You have cared for this patient for the past 6 months and no additional medical records are available. He reports that before your care, his last visit to a physician was 10 years ago. He also reports that he received a tetanus “shot” in the emergency department four years ago because of a laceration. Which of the following is the most appropriate schedule for immunization?

(A) Influenza vaccine only at this visit

(B) Influenza and pneumococcal vaccines at this visit

(C) Influenza vaccine at this visit and pneumococcal vaccine in eight weeks

(D) Pneumococcal vaccine at this visit and influenza vaccine in eight weeks

(E) Don't know

16. A 76-year-old woman has pain in the right hip and is unable to walk without support 3 days after a fall. Passive motion of the right hip produces pain in the groin. Plain radiographs of the pelvis show no abnormalities. The most likely cause of her symptoms is

(A) Muscle strain

(B) Occult fracture of the hip

(C) Osteoarthrosis

(D) Referred pain from the spine

(E) Don't know

17. A frail 70-year-old with early Alzheimer's disease and mild dementia complains of chronic pain. Physical examination is rather unremarkable. X-rays of the lumbar spine show mild changes consistent with osteoarthritis. In consultation, the radiologist suggests that the x-ray findings are minimal and may not account for the pain. The most accurate evidence of the existence of pain and its intensity is:

(A) The nurse's report

(B) The patient's report

(C) Physical examination findings

(D) Specific biological markers of pain

(E) X-ray evidence of osteoarthritis

(F) Don't know

18. A 68-year-old retired male carpenter presents for chronic intermittent low back pain. He rates the pain a 4/10 that is worse with sitting. Radiological studies show minimal degenerative changes of the spine, and magnetic resonance imaging and electromyogram show no evidence of spinal stenosis or nerve root impingement. He takes medications only as a “last resort.” What nonpharmacological treatments may be beneficial?

(A) A conditioning exercise program

(B) Physical therapy

(C) A program of education and cognitive-behavioral therapy

(D) A program of relaxation and message techniques

(E) All of the above

(F) Don't know

Reference [57].
