# Supplementary material for: ChatGPT’s Attitude, Knowledge, and Clinical Application in Geriatrics Practice and Education: Exploratory Observational Study
Source: JMIR Form Res. 2025 Jan 3;9:e63494. doi: 10.2196/63494 (PMC11742095; doi:10.2196/63494)
Supplement: Multimedia Appendix 3 [file formative_v9i1e63494_app3.docx]

**Table 1.** Comparison of geriatrics attitude and knowledge test performance between ChatGPT and trainees.

|  | ChatGPT response 1 | ChatGPT response 2^a^ | ChatGPT response 3 | ChatGPT response 4 | ChatGPT overall response score from 4 prompts | MS^b^ 1^c^ | MS 2 | MS 3 | PGY^d^ 1 | PGY 2 | PGY 3 | GMF^e^ | Overall response score of all trainees |
| --- | --- | --- | --- | --- | --- | --- | --- | --- | --- | --- | --- | --- | --- |
| Total geriatrics attitude mean score^f^ | Did not select any option | 2.9 | 2.4 | 2.7 | 2.7 | 3.9 | 3.7 | 3.6 | 3.4 (3.6) | 3.6 (3.8) | 3.8 (3.7) | 4.1 (4.1) | 3.7 (3.8) |
| Positive attitude mean score^h^ | Did not select any option | 4.5 | 3.8 | 4.0 | 4.1 | —^g^ | — | — | — | — | — | — | — |
| Negative attitude mean score^i^ | Did not select any option | 2 | 1.8 | 1.6 | 1.8 | — | — | — | — | — | — | — | — |
| Geriatrics knowledge mean score^j^ | 12 | 16 | 16 | 15 | 14.7 | 9.9 | 9.5 | 13.6 | 10.4 (13.2) | 11.6 (14.7) | 12 (14.9) | 12 (17.5) | 11.3 (13.3) |

^a^1=*strongly disagree*, 2=*somewhat disagree*, 3=*neutral*, 4=*somewhat agree*, and 5=*strongly agree*.

^b^MS: medical student. MS 1=first-year MS, MS 2=second-year MS, MS 3=third-year MS.

^c^For residents and geriatric medicine fellows, the comparison on geriatrics attitude and knowledge from the validation studies was based on the studies by Reuben et al [49,57] from the same research team. For MSs, the comparison on geriatrics attitude was based on the study by Kishimoto et al [48], and the comparison on geriatrics knowledge was based on a follow-up study in the work by Kishimoto et al [48] because the validation studies only reported a percentage scale that was not reliably converted to the absolute number (–18 to +18). The scores on the follow-up study are also included for comparison in parentheses.

^d^PGY: postgraduate year. PGY 1=first PGY, PGY 2=second PGY, PGY 3=third PGY.

^e^GMF: geriatric medicine fellow.

^f^N=16; score range 1-5; means are for each prompt.

^g^Not applicable.

^h^n=6; score range 1-5; means are for each prompt.

^i^n=10; score range 1-5; means are for each prompt.

^j^Score range –18 to +18; means are for each prompt.
