# Supplementary material for: ChatGPT’s Attitude, Knowledge, and Clinical Application in Geriatrics Practice and Education: Exploratory Observational Study
Source: JMIR Form Res. 2025 Jan 3;9:e63494. doi: 10.2196/63494 (PMC11742095; doi:10.2196/63494)
Supplement: Multimedia Appendix 4 [file formative_v9i1e63494_app4.docx]

**Multimedia Appendix 3.** Comparison of performance on the geriatrics attitude subscales by ChatGPT, trainees, and neurologists.

| Geriatrics attitude scale item | Participants: ChatGPT first response | Participants: ChatGPT second repeat response^a^ | Participants: ChatGPT third repeat response | Participants: ChatGPT fourth repeat response | Participants: ChatGPT responses, from 4 prompts, mean (SD) | Participants: American MS4^b,c^ | Participants: American ED^d^ residents | Participants: Turkish neurologist | Participants: Trainee and neurologist responses, mean (SD) |
| --- | --- | --- | --- | --- | --- | --- | --- | --- | --- |
| Most old people are pleasant to be with. | Only comments; no choice of options | 4 | Comments 3 | 3 | 3.3 | 4.1 | 3.6 | 4.5 | 4.1 |
| *The federal government should reallocate money from Medicare to research on AIDS or pediatric diseases*^e^. | Only comments; no choice of options | 2 | Comments 2 | 3 | 2.3 | 2.2 | 3.7 | 3.6 | 3.2 |
| *If I have the choice, I would rather see younger patients than elderly ones.* | Only comments; no choice of options | 3 | 2 | 3 | 2.7 | 3.3 | 2.8 | 3.4 | 3.2 |
| It is society’s responsibility to provide care for its elderly persons. | Only comments; no choice of options | 5 | 4 | 4 | 4.3 | 4.4 | 4.0 | 4.1 | 4.2 |
| *Medical care for old people uses up too much human and material resources.* | Only comments; no choice of options | 3 | Comments 3 | 3 | 3 | 2.4 | 3.0 | 3.1 | 2.8 |
| *As people grow older, they become less organized and more confused.* | Only comments; no choice of options | 2 | Comments 1 | 2 | 1.7 | 3.1 | 2.8 | 2.2 | 2.7 |
| Elderly patients tend to be more appreciative of the medical care I provide than are younger patients. | Only comments; no choice of options | 4 | Comments 3 | 4 | 3.7 | 3.2 | 3.3 | 3.8 | 3.4 |
| *Taking a medical history from elderly patients is frequently an ordeal.* | Only comments; no choice of options | 2 | Comments 2 | 2 | 2 | 3.2 | 2.4 | 2.3 | 2.6 |
| I tend to pay more attention and have more sympathy towards my elderly patients than my younger patients. | Only comments; no choice of options | 4 | Comments 4 | 4 | 4 | 2.9 | 3.0 | 3.6 | 3.2 |
| *Old people in general do not contribute much to society.* | Only comments; no choice of options | 1 | Comments 1 | 1 | 1 | 1.8 | 4.3 | 3.5 | 3.2 |
| *Treatment of chronically ill old patients is hopeless.* | Only comments; no choice of options | 1 | Comments 1 | 2 | 1.3 | 1.8 | 3.7 | 2.9 | 2.8 |
| *Old persons don’t contribute their fair share towards paying for their health care.* | Only comments; no choice of options | 2 | Comments 3 | 2 | 2.3 | 1.5 | 4.0 | 3.4 | 3.0 |
| *In general, old people act too slow for modern society.* | Only comments; no choice of options | 2 | Comments 1 | 2 | 1.7 | 1.8 | 4.1 | 2.6 | 2.8 |
| It is interesting listening to old people’s accounts of their experience. | Only comments; no choice of options | 5 | Comments 5 | 5 | 5 | 4.3 | —^f^ | 4.1 | 4.2 |
| I feel comfortable working with elderly adults. | Only comments; no choice of options | 5 | Comments 4 | 4 | 4.33 | — | — | — | — |
| *Most elders feel uncomfortable discussing the issue of death and dying with their physicians.* | Only comments; no choice of options | 2 | Comments 2 | 3 | 2.3 | — | — | — | — |
| Positive attitude subscore, mean (SD) of question 1,4,7,9,14,15) from each prompt | Did not select any option | 4.5 | 3.8 | 4 | 4.1 | 3.7 | 3.5 | 4.0 | 3.7 |
| Negative attitude subscore, mean (SD) of question 2,3,5,6,8,10-13, 16 from each prompt | Did not select any option | 2 | 1.8 | 1.6 | 1.8 | 2.1 | 3.1 | 2.7 | 2.6 |

^a^1=*strongly disagree*, 2=*somewhat disagree*, 3=*neutral*, 4=*somewhat agree*, and 5=*strongly agree*.

^b^MS4: fourth-year medical student.

^c^These data are based on published studies [72-74].

^d^ED: emergency department.

^e^Italics indicate negative geriatrics attitudes.

^f^Not applicable.
