# Supplementary material for: ChatGPT’s Attitude, Knowledge, and Clinical Application in Geriatrics Practice and Education: Exploratory Observational Study
Source: JMIR Form Res. 2025 Jan 3;9:e63494. doi: 10.2196/63494 (PMC11742095; doi:10.2196/63494)
Supplement: Multimedia Appendix 5 [file formative_v9i1e63494_app5.docx]

**Multimedia Appendix 4.** Examples of ChatGPT outputs on geriatrics attitude and knowledge questions.

| Prompt^a^ | Original outputs from ChatGPT without any edits | Comments from the author |
| --- | --- | --- |
| “I would like to explore the response to geriatrics attitudes questions. DIRECTIONS: Please use the scale to indicate the degree to which you agree or disagree with each statement. There are no right or wrong answers. The best response is the one that truly reflects your personal opinion. Findings of this study will be reported only on a group basis with no individual names identified. ‘Old people’ and ‘elderly patients’ mentioned in the questions refer to persons aged 65 or older. 1=Strongly Disagree 2=Somewhat Disagree 3=Neutral 4=Somewhat Agree 5=Strongly Agree.  Question 1. Most old people are pleasant to be with.” | “As a text-based AI, I don’t have personal opinions, but I can provide a simulated response based on the scale you provided:  3=Neutral  This response indicates a neutral stance, suggesting that there’s no strong agreement or disagreement with the statement ‘Most old people are pleasant to be with.’ Keep in mind that responses to such statements can vary based on individual experiences and perspectives” | This is 1 of 16 geriatrics attitude questions. This was from the third prompt and output. ChatGPT selected options and provided a rationale for its choice. However, in the first prompt, ChatGPT only generated comments and rational to help you make choice of options but did not select the choice of options. In the other 2 prompts, ChatGPT only selected an option but did not provide a rationale for its choice. |
| “The following items are designed to measure the progressive growth of your knowledge in geriatric medicine. You are therefore not expected to know all the answers until you are ready to graduate from medical school. However, to minimize unfounded guessing, we are using a slightly unusual scoring system. The survey will be scored with a +1 for a correct answer, a −1 for an incorrect answer, and a 0 for the don’t know choice. Please answer ‘Don’t know’ for those items that you truly have no idea of the right answer. DIRECTIONS: PLEASE CIRCLE ‘T’ FOR ‘TRUE,’ ‘F’ FOR ‘FALSE’ OR ‘?’ FOR ‘DON’T KNOW’ ON THE FOLLOWING ITEMS.  Question 1. Memory loss is a normal part of aging. T or F or don’t know” | “False. Memory loss is not considered a normal part of aging. While it’s true that some mild forgetfulness can occur with age, significant memory loss or cognitive decline may be indicative of underlying medical conditions such as dementia or Alzheimer’s disease. Regular forgetfulness is not an inevitable part of the aging process.” | This is 1 of 8 geriatrics true-or-false questions. In 2 prompts, ChatGPT selected an option with a rationale. In the other 2 prompts, ChatGPT provided only a choice without a rationale. |
| “Directions: each of the questions or incomplete statements below is followed by suggested answers or completions. select the one that is best in each case. use the ‘don’t know’ choice when you have no basis for making an educated guess.  Question 9. A 78-year-old nursing home resident has mild dementia associated with Alzheimer’s disease. She is disoriented to time and place but knows family members and regular nurse aides by name. This patient’s capacity to make decisions regarding her health care is best determined by: (A) Mental status test (B) Her ability to understand treatment options (C) Probate court decision (D) Psychiatric examination (E) Don’t know” | “The best choice for determining the capacity of a 78-year-old nursing home resident with mild dementia to make decisions regarding her health care would be: (B) Her ability to understand treatment options.  Assessing an individual’s understanding of treatment options is a key factor in determining their capacity to make informed decisions about their healthcare. It focuses on the specific decision at hand and evaluates the person’s ability to comprehend relevant information, weigh the pros and cons, and communicate their choice.  The other options, such as a mental status test, probate court decision, or psychiatric examination, may not directly address the person’s capacity to make decisions about their healthcare in the context of mild dementia and understanding treatment options.” | This is 1 of 10 geriatrics case questions. In 2 prompts, ChatGPT selected an option with a rationale. In the other 2 prompts, ChatGPT provided only a choice without a rationale. |

^a^Prompts were based on previously published studies [48,49,57].
