# Supplementary material for: ChatGPT’s Attitude, Knowledge, and Clinical Application in Geriatrics Practice and Education: Exploratory Observational Study
Source: JMIR Form Res. 2025 Jan 3;9:e63494. doi: 10.2196/63494 (PMC11742095; doi:10.2196/63494)
Supplement: Multimedia Appendix 6 [file formative_v9i1e63494_app6.docx]

**Multimedia Appendix 5.** ChatGPT output on the polypharmacy vignette about a woman aged 85 years.

| Prompt | Original output from ChatGPT without any edits | Comments from the author |
| --- | --- | --- |
| “The following are 4 general questions:  Which of the following drug (s) is (are) potentially inappropriate medications and should be avoided in older adults? Amiodarone, Glyburide, Valium, Paroxetine, Doxycycline, Benadryl, Doxazosin, Zolpidem” | “All of them except Doxycycline” | Correct |
| “What are potentially inappropriate medications (PIM) in older adults?” | “Potentially Inappropriate Medications (PIM) are medications that may pose a higher risk of adverse events and side effects in older adults compared to younger adults due to age-related changes in the body’s metabolism and elimination processes. PIMs can lead to a range of adverse events, including falls, cognitive impairment, gastrointestinal issues, and more severe complications such as hospitalization. The Beers criteria should be consulted.” | Correct |
| “How to identify potentially inappropriate medications (PIM) in older adults?” | “The most widely used criteria for identifying PIMs in older adults is the Beers Criteria. Other tools and criteria include the Screening Tool of Older Persons' Prescriptions (STOPP), the Medication Appropriateness Index (MAI), and the Drug Burden Index (DBI). When identifying PIMs in older adults, healthcare providers should consider the patient’s medical history, including comorbidities and other medications they are taking, as well as the potential risks and benefits of each medication. They should also consider the patient’s preferences and values when making decisions about medication use. It is important to use a comprehensive approach to medication management, including regular medication reviews and close monitoring for adverse events.” | Correct |
| “What to do with potentially inappropriate medications?” | “Depending on the situation, healthcare providers may take several steps to address PIMs in older adults, including:   1. Discontinuing or reducing the dose of the PIM: If the PIM is not essential for the patient’s health, healthcare providers may consider discontinuing the medication or reducing the dose to minimize the risk of adverse events. 2. Substituting with a safer alternative: In some cases, there may be safer alternatives to the PIM that can be used instead. For example, a medication with a lower risk of side effects or drug interactions may be prescribed. 3. Adjusting the medication regimen: Healthcare providers may adjust the patient’s medication regimen to minimize the risk of adverse events. This may involve changing the timing or frequency of medication administration or adjusting the dose. 4. Educating the patient and family: Patients and their families should be educated about the risks and benefits of the medications they are taking, and the importance of medication adherence and monitoring for adverse events. 5. Regular medication review: Regular medication reviews are essential for identifying new PIMs, monitoring for adverse events, and ensuring that the patient’s medication regimen is appropriate and effective.   It is important to note that the management of PIMs in older adults should be individualized and based on the patient’s unique circumstances and medical history. The goal is to maximize the benefits of medication use while minimizing the risks of adverse events.” | They are possibly appropriate responses. |
| “Any drug-drug interactions for this patient?”^a^ | - “Warfarin and Amiodarone: Amiodarone can increase the effect of warfarin and increase the risk of bleeding. - Furosemide and Spironolactone: Concurrent use of these two drugs may increase the risk of dehydration and electrolyte imbalances. - Lisinopril and Spironolactone: Concurrent use of these two drugs may increase the risk of hyperkalemia. - Carvedilol and Lisinopril: Concurrent use of these two drugs may cause a significant drop in blood pressure. - Glyburide and Amiodarone: Amiodarone can increase the effect of glyburide and increase the risk of hypoglycemia.” | Missed the interaction between levothyroxine and a supplement |
| “Any drug is ineffective for this patient?”^a^ | “It’s difficult to say if any drug is ineffective for this patient without more information about their condition and medical history.” | Missed supplements |
| “She is taking so many medications (called polypharmacy). Any way to reduce the number of her medications?”^a^ | “Some medications may be considered for deprescribing, such as glyburide, which is potentially inappropriate in older adults with diabetes due to its risk of hypoglycemia. Additionally, furosemide may be considered for deprescribing if her fluid status is stable, and her edema has resolved. However, any changes to her medication regimen should be discussed and carefully monitored by her healthcare provider. A comprehensive geriatric assessment may be helpful in identifying appropriate medications and potential targets for deprescribing.” | The prompt question is general. ChatGPT forgot its previous response to the following aforementioned question: **“**What to do with potentially inappropriate medications?” |
| “Any drug-disease interaction for this patient?”^a^ | 1. “Glyburide can cause hypoglycemia, which is a concern for a patient with diabetes. 2. Furosemide and spironolactone can affect electrolyte balance, which can exacerbate heart failure and arrhythmias. 3. Amiodarone can cause pulmonary toxicity, which is a concern for a patient with a history of lung disease. 4. Carvedilol can cause bronchospasm, which is a concern for a patient with a history of lung disease.” | Missed 3 drug-disease interactions, including allopurinol with chronic kidney disease (CKD); carvedilol, spironolactone, lisinopril, and furosemide with orthostatic hypotension; and amiodarone with hypothyroidism |

^a^Specific question based on a geriatrics vignette.
