# Supplementary material for: ChatGPT’s Attitude, Knowledge, and Clinical Application in Geriatrics Practice and Education: Exploratory Observational Study
Source: JMIR Form Res. 2025 Jan 3;9:e63494. doi: 10.2196/63494 (PMC11742095; doi:10.2196/63494)
Supplement: Multimedia Appendix 7 [file formative_v9i1e63494_app7.docx]

**Multimedia Appendix 6.** Fall risk identification and management in a woman aged 82 years.

| Prompt | Original output from ChatGPT without any edits | Comments from the author |
| --- | --- | --- |
| General question: “Risk factors for fall in older adults.” | “Falls are a common problem among older adults and can lead to serious injuries and complications. Some of the risk factors for falls in older adults include:   1. Age: As people age, their balance, vision, and reflexes may decline, making them more susceptible to falls. 2. Medications: Certain medications, such as sedatives, tranquilizers, and blood pressure medications, can increase the risk of falls by causing dizziness or lightheadedness. 3. Chronic conditions: Chronic conditions such as arthritis, Parkinson’s disease, stroke, and dementia can increase the risk of falls by affecting balance, mobility, and coordination. 4. Muscle weakness and poor balance: Muscle weakness, especially in the legs, and poor balance can increase the risk of falls. 5. Vision problems: Poor vision or eye conditions such as cataracts, glaucoma, or macular degeneration can increase the risk of falls. 6. Environmental hazards: Hazards in the home such as poor lighting, loose rugs or carpets, and clutter can increase the risk of falls. 7. Lack of physical activity: Lack of physical activity and exercise can lead to muscle weakness, poor balance, and decreased flexibility, all of which increase the risk of falls. 8. Poor nutrition: Poor nutrition can lead to muscle weakness and fatigue, which can increase the risk of falls. 9. Alcohol use: Alcohol use can affect balance and coordination, increasing the risk of falls. 10. History of falls: Having a history of falls increases the risk of future falls.   It’s important for older adults and their caregivers to be aware of these risk factors and take steps to minimize them to prevent falls and maintain independence.” | ChatGPT provided a comprehensive summary of fall risks. However, a few risk factors were missed, such as fear of falls and cardiovascular diseases. |
| Pretest 1: “When an older person falls, should you ask the following questions (one or more or none)? the circumstances of fall, mental status, medication changes, vision change, drinking.” | “Yes, it is recommended to ask these questions when an older person falls:   1. Circumstances of fall: It’s important to ask about the circumstances of the fall to determine what may have caused it, such as environmental hazards or a sudden change in balance. 2. Mental status: It’s important to assess the older person’s mental status to determine if they are alert and oriented or if they have suffered a head injury or other trauma that may have affected their cognition. 3. Medication changes: It’s important to ask about any recent changes in medication, as some medications can increase the risk of falls. 4. Vision changes: It’s important to ask about any recent changes in vision, as vision problems can increase the risk of falls. 5. Drinking: It’s important to ask about alcohol use, as alcohol can affect balance and coordination and increase the risk of falls.   By asking these questions, healthcare providers or caregivers can identify potential risk factors for falls and take steps to prevent future falls and promote safety for the older person.” | ChatGPT provided fairly good recommendations. |
| Pretest 2: “Which of the following illnesses is a fall risk (one or more or none)? Pneumonia, Parkinson’s disease, Diabetes mellitus, Dementia, Orthostatic hypotension” | “Parkinson’s disease, dementia, and orthostatic hypotension are all illnesses that can increase the risk of falls in older adults.  Parkinson’s disease can cause movement and balance problems, making it more difficult for people with this condition to stay upright and avoid falls.  Dementia can cause confusion, disorientation, and poor judgment, increasing the risk of falls.  Orthostatic hypotension is a condition in which blood pressure drops when a person stands up, causing dizziness and lightheadedness, which can lead to falls.  Pneumonia and diabetes mellitus are not typically considered illnesses that increase the risk of falls in older adults. However, diabetes can cause neuropathy or nerve damage, which can lead to a loss of sensation in the feet and legs, increasing the risk of falls.” | ChatGPT missed that diabetes mellitus and pneumonia are fall risk factors in older adults. |
| Pretest 3: “Which of the following medications(s) is (are) Not associated with an increased fall risk (one or more)? Levothyroxine Diazepam Oxycodone Amitriptyline” | “Levothyroxine is not typically associated with an increased risk of falls.  Diazepam, oxycodone, and amitriptyline are medications that can increase the risk of falls in older adults. Diazepam is a benzodiazepine used to treat anxiety and can cause dizziness and drowsiness, which can increase the risk of falls. Oxycodone is an opioid pain medication that can cause dizziness, drowsiness, and impaired coordination, increasing the risk of falls. Amitriptyline is a tricyclic antidepressant that can cause dizziness, confusion, and impaired coordination, increasing the risk of falls.” | Correct |
| Pretest 4: “Which one(s) of the following is (are) true (one or more or none)? An elderly person may become so fearful of falling that they restrict his/her mobility. Most falls are associated with significant injuries. Falls is a leading cause of accidental death in older population. About 5% community-dwelling elderly person falls each year. Survivors of fall-related hip fracture are rarely institutionalized. Exercise improves function and reduces fall risk and injurious falls.” | “The following statements are true:   1. An elderly person may become so fearful of falling that they restrict his/her mobility: Fear of falling can lead older adults to limit their activities and avoid going out, which can lead to a decline in physical activity, further weakening muscles and increasing the risk of falls. 2. Exercise improves function and reduces fall risk and injurious falls: Exercise can improve balance, strength, and flexibility, reducing the risk of falls and injuries from falls. 3. About 5% community-dwelling elderly person falls each year: Falls are common among older adults, and approximately 5% of community-dwelling older adults fall each year.   The following statements are false:   1. Most falls are associated with significant injuries: While falls can cause serious injuries, most falls do not result in significant injuries. 2. Falls are a leading cause of accidental death in older population: Falls are a significant cause of injury among older adults, but they are not a leading cause of accidental death. Other causes, such as motor vehicle accidents and accidental poisoning, are more common causes of accidental death. 3. Survivors of fall-related hip fracture are rarely institutionalized: Fall-related hip fractures can be serious and can lead to hospitalization and long-term care. Many older adults who suffer from a hip fracture require institutionalized care for rehabilitation and support.” | ChatGPT made 2 mistakes. First, it is false that 5% of community-dwelling older persons fall each year. ChatGPT responded that it was true. In fact, one-third of community-dwelling older adults fall every year. Second, it is true that falls are a leading cause of accidental death in the older population. ChatGPT responded that it was false. |
| Pretest 5: “Which of the following is NOT an environmental fall risk? (only pick one answer) Throw rug, freshly waxed kitchen floor, Grab bars, Electrical cord lying on the floor” | “Grab bars are not an environmental fall risk. In fact, grab bars can be helpful in preventing falls by providing support and stability when moving around in the bathroom or shower.  Throw rugs, freshly waxed kitchen floors, and electrical cords lying on the floor are all examples of environmental fall risks. Throw rugs can slide or bunch up, causing someone to trip and fall. Freshly waxed floors can be slippery and cause a loss of balance. Electrical cords lying on the floor can create a tripping hazard.” | Correct |
| Prompt 1: “List all potential fall risk factors for this patient.” | - “History of multiple falls - Previous right hip fracture - Diagnosis of hypertension, coronary heart disease, and knee arthritis - Not using assistive devices (cane and walker) frequently - Caregiving responsibilities for her husband - Medications that may increase fall risk (Doxazosin, Diltiazem)” | ChatGPT identified all fall risk factors except potential home environment factors for this patient. |
| Prompt 2: “What are three fall screening questions you need to ask for elderly patients in an outpatient setting based on 2010 fall prevention guideline you want to follow for this patient?” | 1. “Have you fallen in the past year? 2. Do you feel unsteady when standing or walking? 3. Do you worry about falling?” | In total, 2 out of 3 responses were consistent with the 2010 fall prevention guidelines (presented with acute fall was missing). However, 3/3 responses were consistent with the 2019 CDC^a^ algorithm for fall risk screening, assessment, and intervention in older patients (STEADI^b^). |
| Prompt 3: “Which one of three pathways in Figure 1 from 2010 fall prevention guideline you want to follow for this patient?” | “The multifactorial fall risk assessment pathway.” | Correct |
| Prompt 4: “List all physical examinations that you think they are important to perform for this patient.” | - “Orthostatic blood pressure measurements - Gait and balance assessment - Visual acuity test - Neurological examination - Examination of lower extremities for strength, range of motion, and joint pain - Evaluation of feet and footwear” | ChatGPT missed several physical examinations, including checking the ear and hearing, the heart, and the lungs. |
| Prompt 5: “List your recommendations as many as you think that could help her to prevent her falling.” | - “Encourage the use of assistive devices (cane and walker) - Review and adjust medications that may increase fall risk - Evaluate her caregiving responsibilities and provide resources for support - Recommend exercises to improve balance and strength - Provide education on fall prevention strategies (e.g., avoiding throw rugs, wearing appropriate footwear, using handrails) - Consider a referral to physical therapy for additional fall prevention interventions.” | ChatGPT provided a good summary of recommendations for this patient. However, ChatGPT missed some other recommendations which could be beneficial for this patient, including vitamin D and calcium supplements and tai chi exercises. |

^a^CDC: Centers for Disease Control and Prevention.

^b^STEADI: Stopping Elderly Accidents, Deaths, and Injuries.
